# Supplementary material for: PP2A and GSK3 act as modifiers of FUS-ALS by modulating mitochondrial transport
Source: Acta Neuropathol. 2024 Feb 16;147(1):41. doi: 10.1007/s00401-024-02689-y (PMC10873455; doi:10.1007/s00401-024-02689-y)
Supplement: Supplementary file 1 — Supplementary file1 (PDF 51134 KB) [file 401_2024_2689_MOESM1_ESM.pdf]

Supplementary material to:

PP2A and GSK3 act as modifiers of FUS-ALS by modulating mitochondrial transport

Paraskevi Tziortzouda[1,2], Jolien Steyaert[1,2], Wendy Scheveneels[1,2], Adria Sicart[1,2], Katarina Stoklund Dittlau[1,2], Adriana Margarida Barbosa Correia[1,2,3], Thibaut Burg[1,2], Arun Pal[4,5], Andreas Hermann[5,6,7,8], Philip Van Damme[1,2,9], Thomas G Moens[1,2,*] & Ludo Van Den Bosch[1,2,*]

[1]Department of Neurosciences, Experimental Neurology and Leuven Brain Institute (LBI), KU Leuven - University of Leuven, Leuven, Belgium

[2]Center for Brain & Disease Research, Laboratory of Neurobiology, VIB, Campus Gasthuisberg, O&N5, Herestraat 49, PB 602, 3000 Leuven, Belgium

[3]Instituto Superior Técnico - Universidade de Lisboa, Av. Rovisco Pais, 1049-001 Lisboa, Portugal

[4]Dresden High Magnetic Field Laboratory (HLD-EMFL), Helmholtz-Zentrum Dresden Rossendorf, D-01328 Dresden, Germany

[5]Division of Neurodegenerative Diseases, Department of Neurology, Technische Universität Dresden, D-01307, Dresden, Germany

[6]Translational Neurodegeneration Section “Albrecht Kossel”, Department of Neurology, University Medical Center Rostock, University of Rostock, D-18147 Rostock, Germany

[7]Deutsches Zentrum fur Neurodegenerative Erkrankungen (DZNE) Rostock/Greifswald, D-18147, Rostock, Germany

[8]Center for Transdisciplinary Neurosciences Rostock (CTNR), University Medical Center Rostock, University of Rostock, D-18147, Rostock, Germany

[9]Department of Neurology, University Hospitals Leuven, Leuven, Belgium

*Corresponding authors

Thomas G Moens, thomas.moens@kuleuven.be, TEL: +32 16 33 06 81, FAX: +32 16 37 25 34

Ludo Van Den Bosch, ludo.vandenbosch@kuleuven.be, TEL: +32 16 33 06 81, FAX: +32 16 37 25 34

Supplementary Figures and Tables

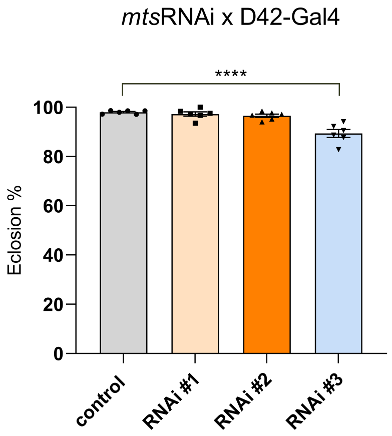

Fig. S1 *mts* RNAi line #3 leads to a lower eclosion percentage due to inherent toxicity. *mts* RNAi lines shown in Fig. 1c were crossed to the D42-Gal4 driver line without FUS expression and eclosion was assessed. Knockdown of *mts* using line #3 causes a significant reduction in eclosion. (N=6 crosses/condition) Statistical comparisons between control and RNAi conditions were determined using one-way ANOVA with Sidak's multiple comparisons test. ****p< 0.0001

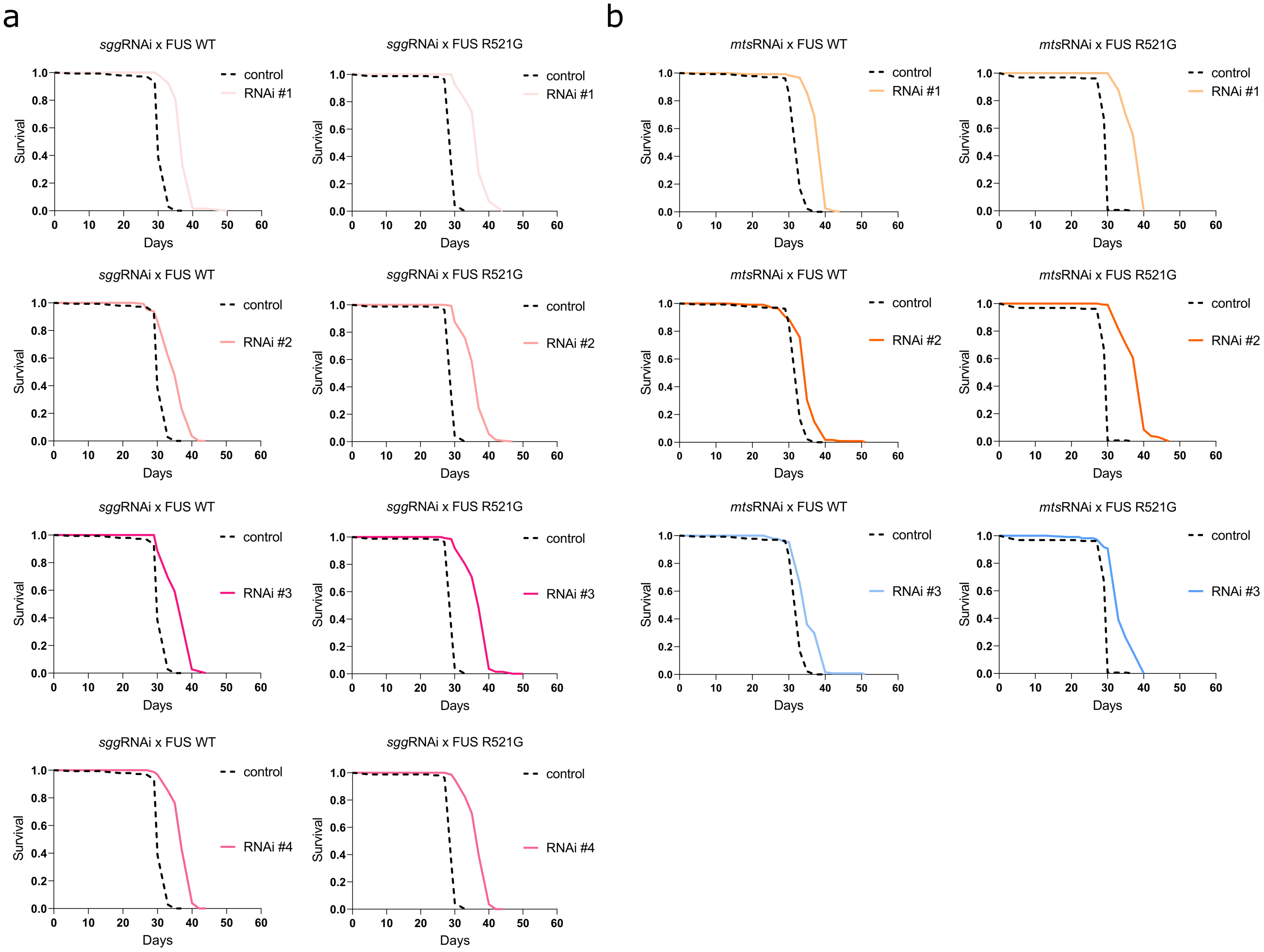

Fig. S2 *sgg* and *mts* inhibition extend the lifespan of the FUS fly. a. Detailed graphs presenting the extension of the lifespan that each *sgg* RNAi line induces. The dashed line indicates the healthy control and the colored line indicates the respective *sgg* RNAi line. **b.** Detailed graphs presenting the extension of the lifespan that each *mts* RNAi line induces. The dashed line indicates the healthy control and the colored line indicates the respective *mts* RNAi line. (log-rank tests, see Table S2 for statistical information)

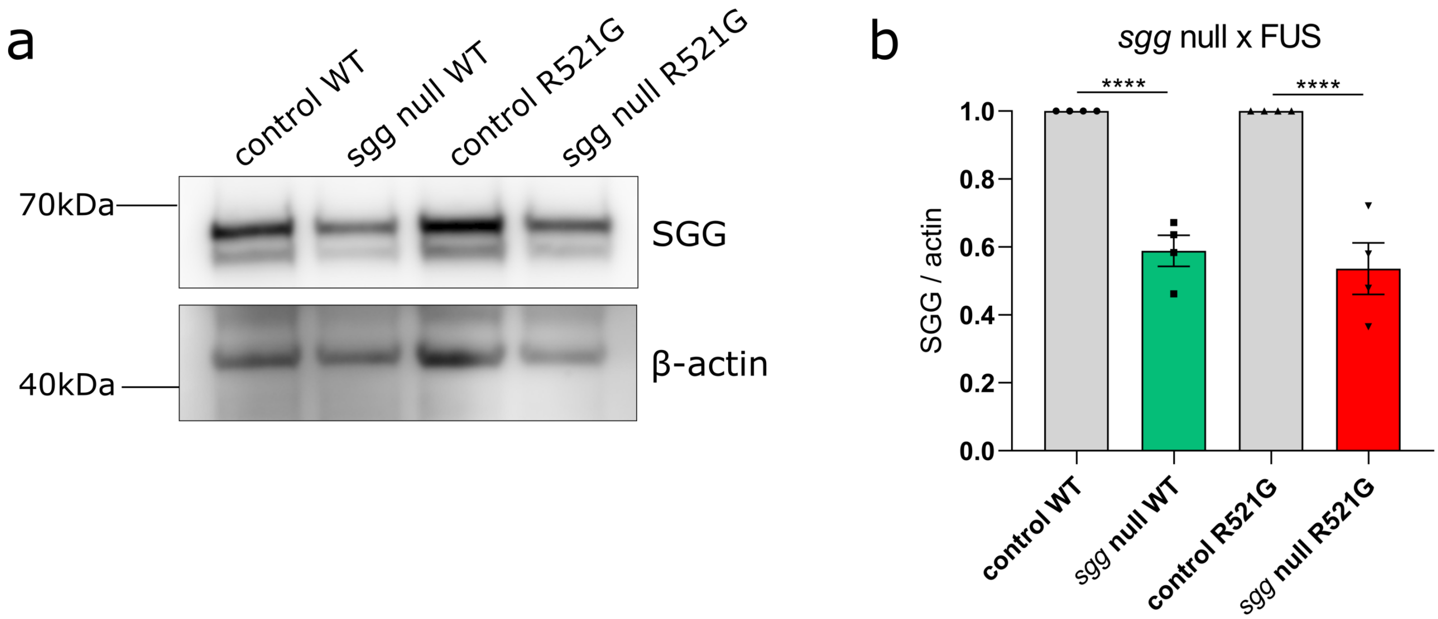

Fig. S3 ***sgg*** **null mutant reduces the levels of sgg protein by ~50%. a.** Western blot for sgg protein levels in *sgg* null mutant line. Beta-actin serves as loading control. (N=3) **b.** Quantification of panel a shows that the *sgg* null mutant line induces ~50% reduction of sgg protein levels. (N=3, mean ± SEM, one-way ANOVA with Sidak's multiple comparisons test) ****p< 0.0001

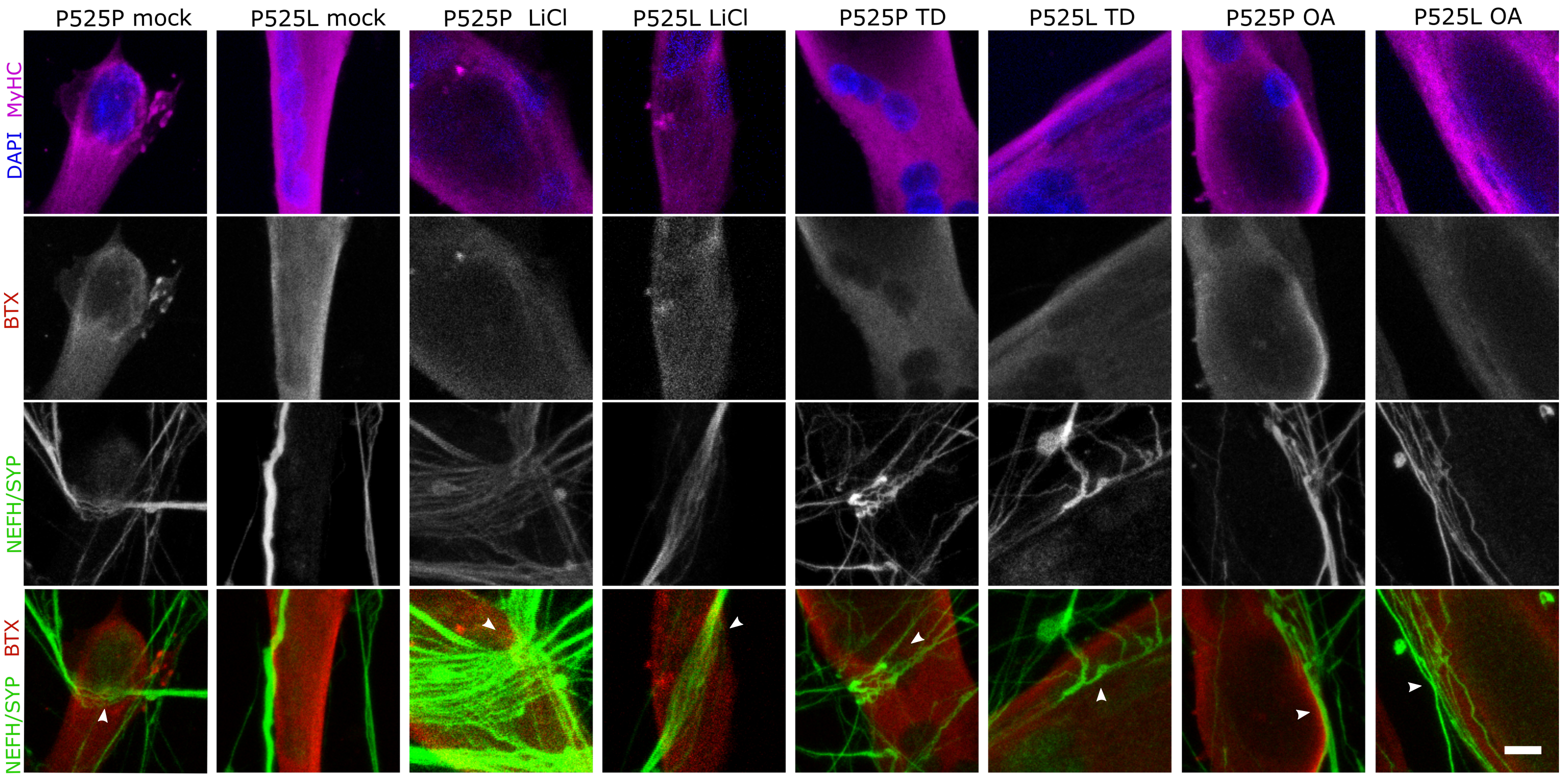

Fig. S4 PP2A and GSK3 inhibition improve FUS-induced NMJ impairments. Additional representative images of all the conditions with and without pharmacological treatments. The quantification of the NMJ-like structures is based on the neurite/presynaptic marker morphology and/or on Btx (red)-SYP/NEFH (green) co-localization per myotube. The DAPI and myosin (MyHC) channels are merged, demonstrating the multi-nucleated myoblast, while the bungarotoxin and synaptophysin channels are presented alone, and finally merged to indicate the formation of the NMJ. Scale bar = 10μm

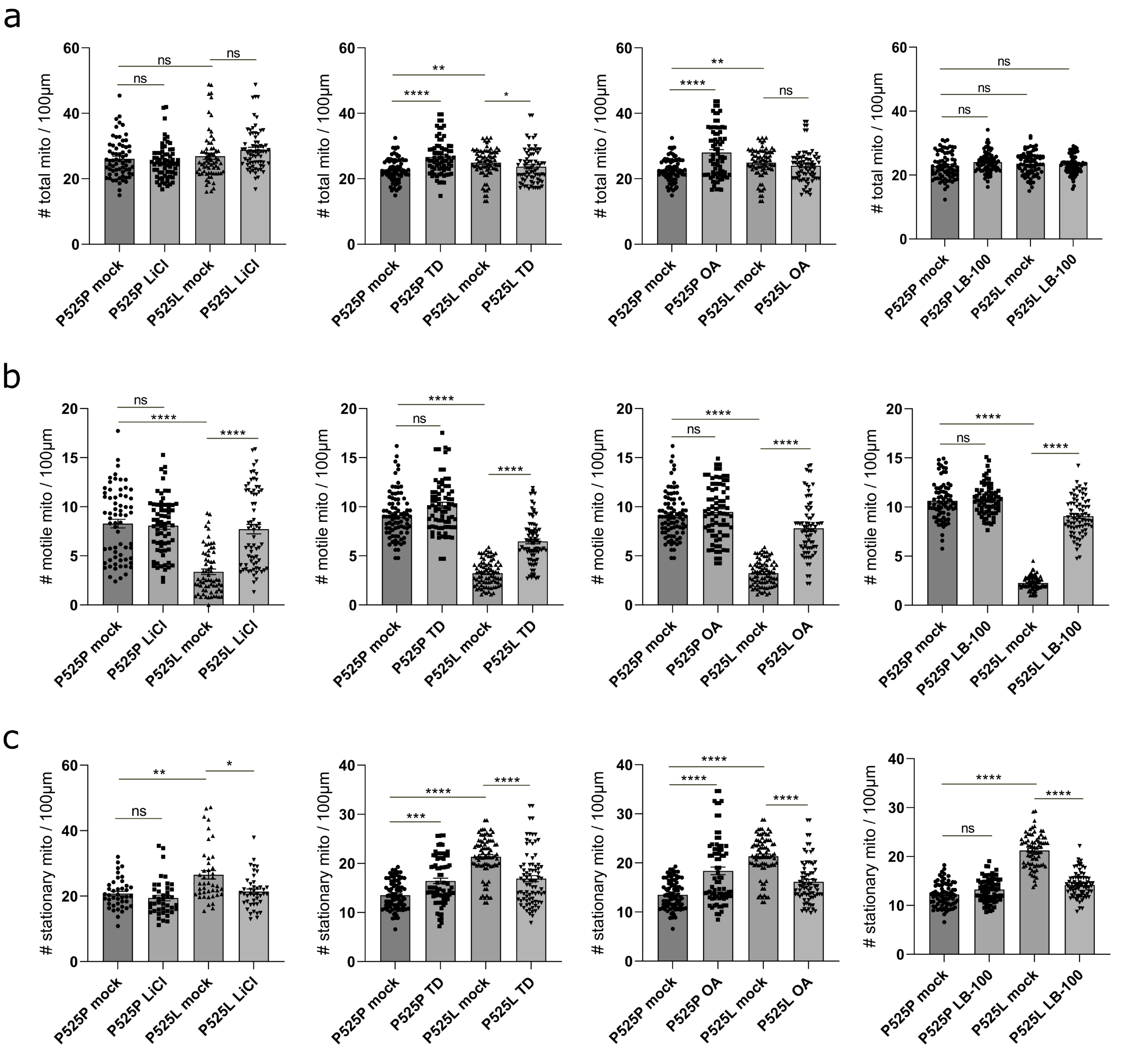

Fig. S5 Additional data on the effect of LiCl, TD, OA and LB-100 on mitochondrial transport in sMNs. a to c. Quantification of total (a), motile (b) and stationary (c) mitochondria normalized to 100µm neurite length. Total mitochondria are calculated as the sum of motile and stationary events. Data are represented as mean ± SEM, N=3 independent differentiations, Kruskal-Wallis with Dunn's multiple comparisons test. ****p< 0.0001, ***p<0.001, **p<0.01, *p<0.05, ns= not significant

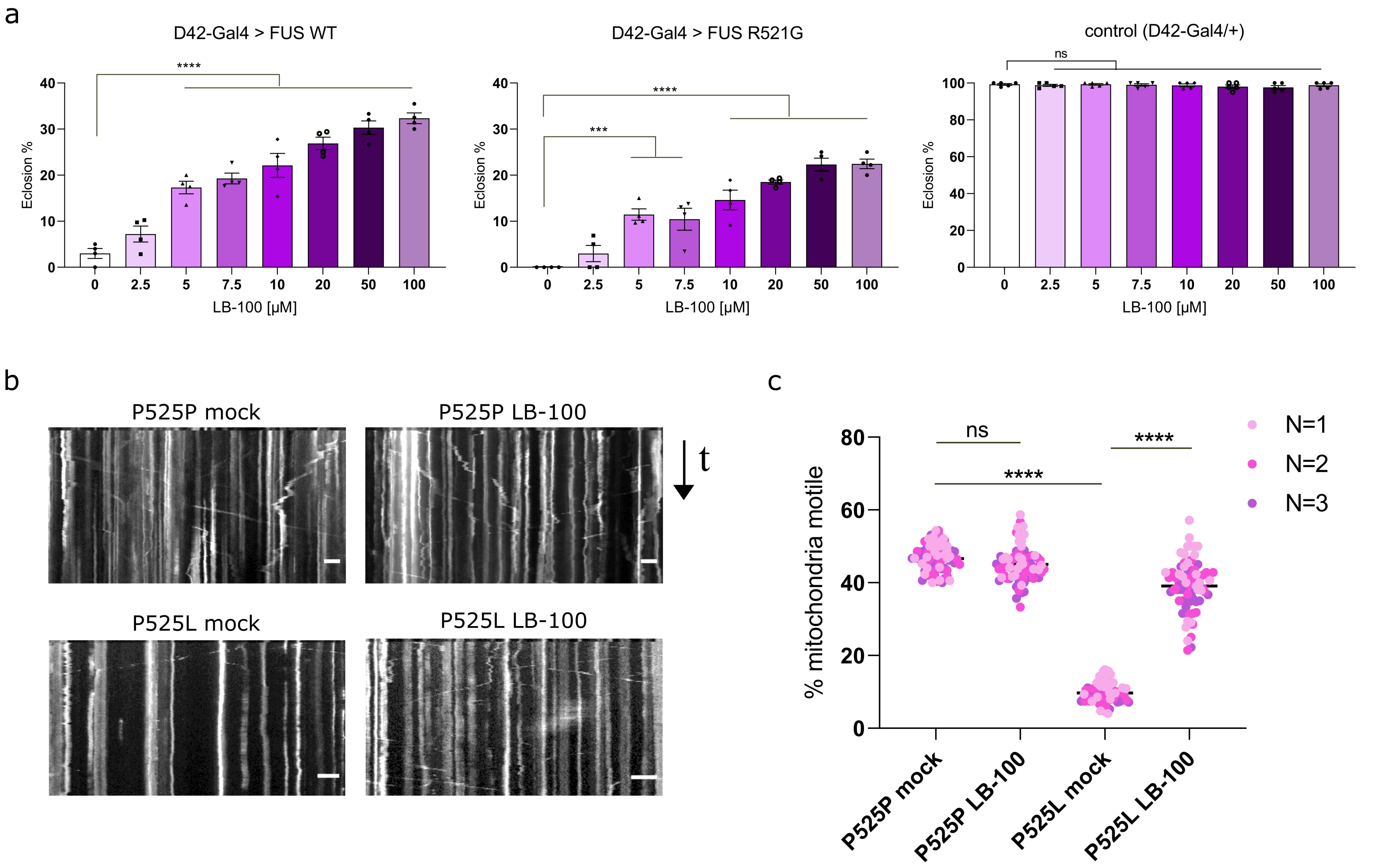

Fig. S6 PP2A pharmacological inhibition by LB-100 rescues phenotypes in FUS flies and patient iPSC-derived motor neurons. a. Pharmacological inhibition of PP2A by LB-100 rescues the eclosion phenotype in FUS flies. D42-Gal4/+ serves as a control. (N=4 or N=5 crosses/condition). Statistical comparisons between untreated and treated conditions were determined using one-way ANOVA with Sidak's multiple comparisons. ****p<0.0001, ***p<0.001, ns = not significant **b.** Example kymographs (time-distance plots) of mitochondria (MitoTracker Green) after treatment of 30-day old P525P isogenic control and P525L mutant motor neurons with 1µM LB-100 for 48h. Scale bars 30µm **c.** Percentage of mitochondria that are motile in the motor neurons (day30) comparing isogenic control and mutant with or without LB-100. Data shown as Grand Mean; N=3 differentiations; Kruskal-Wallis with Dunn's multiple comparisons test. ****p<0.0001, ns = not significant

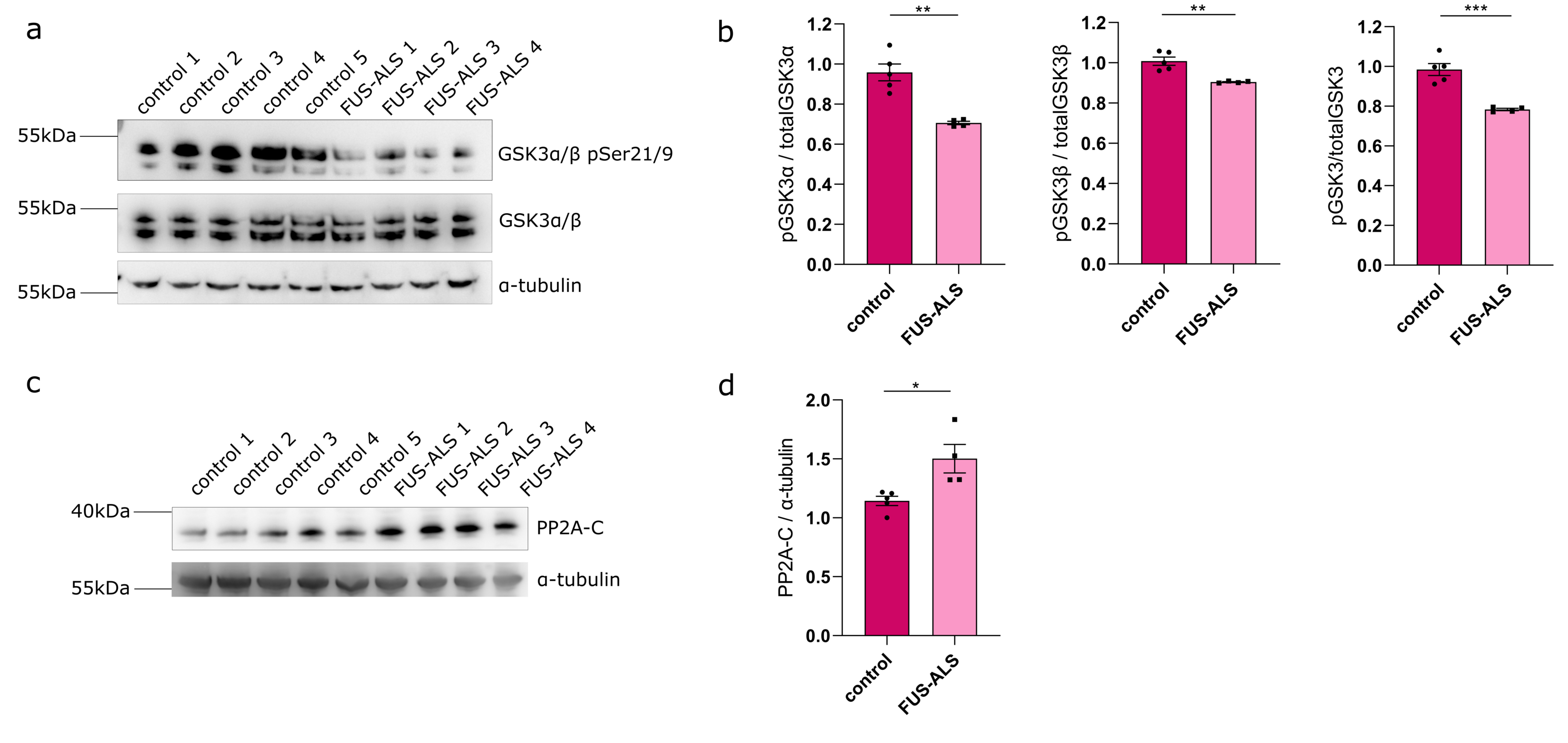

Fig. S7 GSK3 is hyperactive in a FUS-ALS mouse model due to reduced inhibitory phosphorylation. a. Western blot for GSK3α/β pSer21/9 in the spinal cord of 4 FUS mice and 5 healthy controls. Ser21/9 inhibitory phosphorylation of GSK3α/β appears reduced in the FUS mice compared to control mice, while the total levels of GSK3α/β remain unaltered. Alpha-tubulin serves as a loading control. **b.** Quantification of the western blot of panel a shows reduced Ser21 and Ser9 inhibitory phosphorylation for GSK3 α and β respectively. **c.** Western blot for PP2A-C in the spinal cord of 4 FUS mice and 5 healthy controls. PP2A-C appears increased in the FUS mice compared to control mice. Alpha-tubulin serves as a loading control **d.** Quantification of the western blot of panel c shows increased PP2A-C in FUS mice. Mean ± SEM, unpaired t-test, *p<0.05, **p<0.01, ***p<0.001

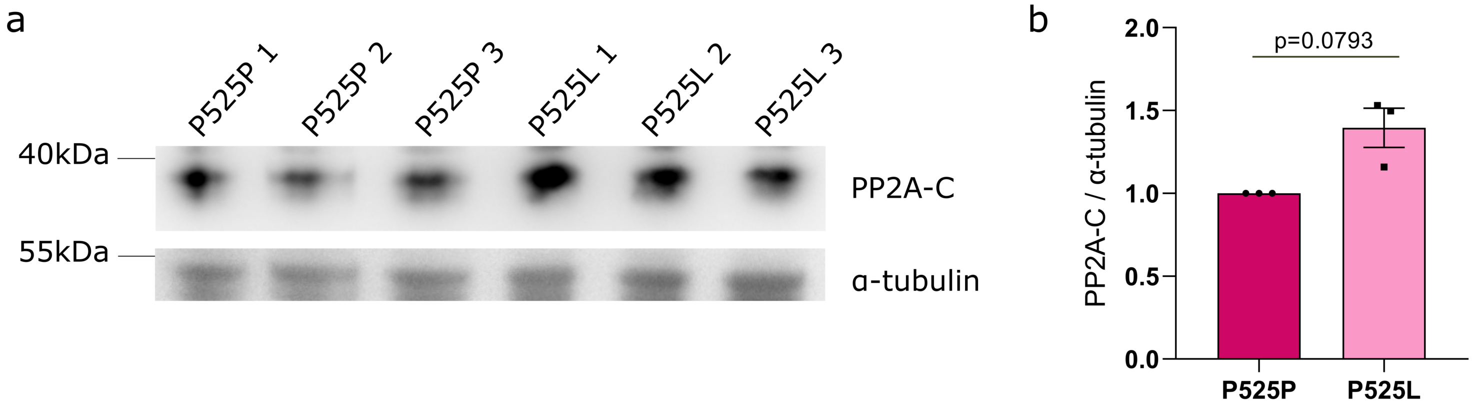

Fig. S8 PP2A-C is elevated in FUS patient iPSC-derived motor neurons. a. Western blot for PP2A-C in FUS iPSC-derived motor neurons from FUS-P525L patients and in their CRISPR-corrected P525P isogenic controls. The numbers 1, 2 and 3 represent three independent differentiations. Alpha-tubulin serves as a loading control. **b.** Quantification of the western blot of panel a shows increased PP2A-C protein levels in P525L mutant motor neurons. (N=3, mean ± SEM, two-tailed paired t-test) p=0.0793

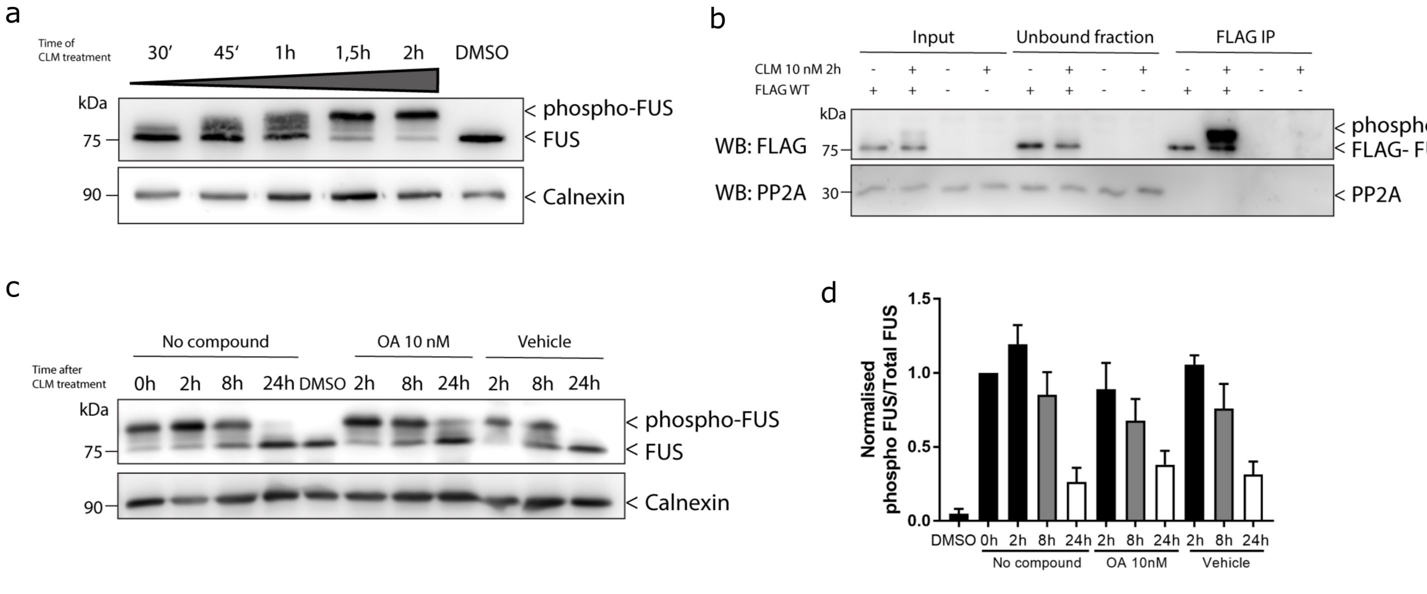

Fig. S9 PP2A is an upstream suppressor of FUS toxicity. a. FUS phosphorylation by 10nM CLM treatment is examined at different time points. After 45 min of CLM treatment, phospho-FUS was observed and after 2 h of CLM treatment, endogenous FUS was almost completely phosphorylated. **b.** Lysates from FLAG-WT hFUS-expressing HEK293T cells treated with 10nM CLM were immunoprecipitated using an anti-FLAG antibody. No interaction between FUS and PP2A was observed. **c.** HEK293T cells treated with 10nM CLM showed almost complete dephosphorylation after 24 h (no compound). PP2A inhibition with 10nM OA could not affect the dephosphorylation rates of FUS. **d.** Quantification of the western blot of panel c.

Table S1: Overview of the candidate modifiers that resulted from the genetic screen.

	Modifying gene	CG number	Human orthologue	Rescue		RNAi lines tested	RNAi lines positive	Null Mutants		Null mutants tested	Null mutants positive
				Female D42/R521G (%)	Male D42/R521G (%)			Female D42/R521G (%)	Male D42/R521G(%)		
Unbiased approach	NaCh	CG8178	SCNN1G	9	-	1	1				
	CG34458	CG34458	CSTG	-	4	1	1				
	Herp	CG14536	HERPUD2	1	3	2	2	-	-	2	0
	Mts	CG7109	PPP2CA	8	1	1	1	12	0	4	2
	CG7231	CG7231	FAM151B	0	3	2	1	7	0	2	1
	Sirup	CG7224	SDHAF4	5	2	1	1	1	0	1	0
	R2d2	CG7138	STAU2	4	4	2	1	4	0	2	1
	SmE	CG18591	SNRPE	5	5	2	1	4	0	1	1
	CG31915	CG31915	COLGALT1	1	0	2	1	85	40	1	1
	CG7277	CG7277	COQ6	0	7	1	1	22	0	1	1
	qkr54B	CG4816	KHDRBS1	1	-	2	1	55	27	1	1
	NT5E-2	CG30104	NTE	1,4	-	2	1			2	0
	Patronin	CG33130	CAMSAP2	13	13	2	1	17	4	3	1
	CG6568	CG6568	unknown	1	-	1	1	3	2	1	1
	l(2)k01209	CG4798	UCKL1	3	-	1	1	-	-	3	0
	CG18467	CG18467	GID8	2	-	1	1				
	FMR1	CG6203	FMR1	3	3	5	3	5	6	2	2
	Sgg	CG2621	GSK3B	63	45	5	2	7	-	3	1
Literature	HRB87F	CG12749	HNRNPA2B1	2	-	1	1	4	-	1	1
	HRB27C	CG10377	DAZAP1	3	6	2	2	50	64	2	1
	Parp	CG40411	PARP1	17	4	2	1				
	Sgg	CG2621	GSK3B	63	45	5	2	7	-	3	1
	qkr54B	CG4816	KHDRBS1	1	-	2	1	55	27	1	1
	FMR1	CG6203	FMR1	3	3	5	3	5	6	2	2

Table S2: The statistical information of the RNAi lifespans (from Fig.2).

	Experiment	Condition	N (number of flies)	Median lifespan (days)	P value
sggRNAi x FUS	WT FUS	control	152	29.5	-
		RNAi #1	149	36.0	5.24807E-58
		RNAi #2	151	34.0	3.62347E-30
		RNAi #3	155	36.0	5.57358E-39
		RNAi #4	156	36.0	2.92332E-54
	R521G FUS	control	160	28.0	-
		RNAi #1	148	36.0	3.37108E-64
		RNAi #2	149	36.0	4.90037E-60
		RNAi #3	148	36.0	1.99196E-60
		RNAi #4	146	36.0	6.91336E-65
mtsRNAi x FUS	WT FUS	control	142	31.5	-
		RNAi #1	134	38.5	3.00091E-48
		RNAi #2	126	34.0	3.87413E-17
		RNAi #3	139	34.0	1.09269E-18
	R521G FUS	control	158	29.5	-
		RNAi #1	128	38.5	1.05996E-52
		RNAi #2	139	38.5	1.50108E-56
		RNAi #3	128	31.5	3.0097E-38

Table S3: The statistical information of the null mutant lifespans (from Fig.2).

	Experiment	Condition	N (number of flies)	Median lifespan (days)	P value
sgg null x FUS	WT FUS	control	137	31.5	-
		sgg null	145	52.5	1.83106E-58
	R521G FUS	control	138	35.0	-
		sgg null	142	48.0	4.03951E-52
mts null x FUS	WT FUS	control	137	31.5	-
		mts null	140	41.0	6.4319E-26
	R521G FUS	control	138	35.0	-
		mts null	150	38.5	6.63065E-15

Table S4: The statistical information of p[Khc+] lifespans (from Fig.8).

	Experiment	Condition	N (number of flies)	Median lifespan (days)	P value
p[Khc+] x FUS	WT FUS	control	144	36.0	-
		p[Khc+]	123	51.0	5.76056E-26
	R521G FUS	control	145	30.5	-
		p[Khc+]	137	48.5	2.01433E-51

Table S5: Primary antibody overview.

Antibody	Dilution	Company	Application	Cat No
Rabbit anti-FUS	1:1000	Bethyl Laboratories	WB	A300-293AM
Mouse anti-beta Actin	1:1000	Abcam	WB	ab8224
Rabbit FUS/TLS Polyclonal	1:500	Proteintech	ICC	11570-1-AP
Goat anti-Choline Acetyltransferase (ChAT)	1:500	Sigma-Aldrich	ICC	AB144P
Mouse anti-beta III Tubulin antibody	1:500	Abcam	ICC	ab7751
Rabbit anti-Neurofilament heavy polypeptide (NEFH)	1:1000	Abcam	ICC	ab8135
Rabbit anti-synaptophysin (SYP)	1:1000	Cell Signaling	ICC	5461S
Mouse anti-myosin heavy chain (MyHC)	1:20	In house, SCIL	ICC	-
Rabbit phospho-GSK-3α/β (Ser21/9)	1:500	Cell Signaling	WB	9331S
Mouse Anti-Glycogen Synthase Kinase 3 (GSK3) (Drosophila)	1:500	US Biological	WB	G8170-40
Mouse anti-α-Tubulin	1:5000	Sigma Aldrich	WB	T6199
Mouse anti-Kinesin Antibody, light chain	1:500	Sigma Aldrich	WB	MAB1617
Mouse kinesin heavy chain, head region (SUK4)	1:500	DSHB	WB	AB_528326
Rabbit Anti-KLC1 S460p	1:500	In house, Chris Miller lab	WB	-
Rabbit Calnexin	1:2000	Enzo Life Sc	WB	ADI-SPA-860-D
Mouse anti-PP2A-C	1:1000	In house, Veerle Janssens lab	WB	-

Table S6: Secondary antibody overview.

Antibody	Dilution	Company	Application	Cat No
Alexa FlourTM IgG (H+L) 488 donkey-anti-rabbit	1:1000	Thermo Fisher Scientific	ICC	AB_2535792
Alexa FlourTM IgG (H+L) 555 donkey-anti-goat	1:1000	Thermo Fisher Scientific	ICC	AB_2535853
Alexa FlourTM IgG (H+L) 647 donkey-anti-mouse	1:1000	Thermo Fisher Scientific	ICC	AB_162542
α-bungarotoxin (Btx) Alexa FlourTM 555	1:1000	Thermo Fisher Scientific	ICC	AB_2617152
Goat anti-Mouse IgG (H+L) Secondary Antibody, HRP	1:1000	Thermo Fisher Scientific	WB	62-6520
Goat anti-Rabbit IgG (H+L) Secondary Antibody, HRP	1:1000	Thermo Fisher Scientific	WB	31460
